# Principles of Human Physiology, with Their Chief Applications to Pathology, Hygiene, and Forensic Medicine

**Published:** 1847-01

**Authors:** 


Art. VIII.—*Principles of Human Physiology, with their chief applications to Pathology, Hygiene, and Forensic Medicine.* By W. B. Carpenter, m.d. f.r.s., Fullerian Professor of Physiology to the Royal Institution of Great Britain. Third Edition, with two copper-plates and 175 woodcuts.—*London*, 1846. 8vo, pp. 776.

As twice before, on the publication of the first and second editions, we had occasion to notice this incomparable work, we shall at this time content ourselves with simply calling the attention of our readers to the new edition, and stating, in a few words, some of the more important additions we have observed in turning over its well-filled pages. We may say generally of this as of the other works of Dr. Carpenter, that the reader may safely trust to the author's industry and quick-sightedness for supplying him with every novelty relating to his subject up to the day of publication. Nothing escapes his observation and his grasp, and few things are unimproved by his handling—*nihil tetigit quod non ornavit.* In so vast a collection of facts, in such a voluminous code of doctrines, it would be difficult, had we both space and time at command, to give even an outline of the numerous and important improvements, both in the way of additions and alterations introduced into the present edition. The following are some of the novelties (not a few of which are either wholly or in part original) that attracted our notice in comparing the new volume with the old. We put them down in the order of the pages, and we have to apologise both to the author and the reader for the dry index-like form in which we present them:

Application of the simple phenomena of cell-growth in the lowest plants to an explanation of similar process in animals. (pp. 87-9).

Clearer view of the two elements of human hair obtained by comparative inquiries into the structure of hair in other animals. (p. 121.)

Additional light thrown on the structure of bone by the author's investigations on shells, &c., of invertebrata, which show that a skeleton may be formed by the calcification of *fibres* or of *cells*, both of which modes are employed in the production of bone. (p. 141 et seq.)

Mr. Quekett's observations on differences in size and form of osseous lacunæ characteristic of different groups of animals. (p. 143.)

Von Bibra's analyses of bone. (p. 146.)

Dr. Sharpey's researches on the development of bone in membrane by calcification of the fibre, confirmed by the author. (p. 147.)

Professor Owen's researches into the early development of tooth structures. (pp, 157-61.)

Ultimate structure of muscular fibre. (p. 176.)

Clear distinction of the *animal* functions as *destructive* in their exercise, and of the *vegetative,* as *reparative* and *preservative* (Liebig had announced this as true only of muscular action). (pp. 201-2.)

Improvements in the arrangement of the subjects in the chapter on the nervous system. Fuller consideration of sensory ganglia and actions of nerves connected with them.

General sketch of the mental powers. (pp. 371-2.)

Fuller consideration of muscular tonicity. (p. 446.)

Various recent researches on digestion, showing the destination of different articles of aliment more clearly. (pp. 510-11.)

Fuller consideration of the process of sanguification. Chap. XI.

Improved mode of viewing the character of the spleen and other vascular glands. (pp. 520-27.)

Chemical novelties regarding the constitution of the blood and process of respiration.

Goodsir's researches on secretion. (p. 633.)

In conclusion, we can only reiterate our former high appreciation of this work and recommend it in the most earnest manner to every student and lover of the highest and most interesting department of medical science.

